# Birth defects and associated antenatal care factors related with 1st trimester of pregnancy

**DOI:** 10.1093/eurpub/ckac130.248

**Published:** 2022-10-25

**Authors:** I Zile-Velika, I Ebela, I Rumba-Rozenfelde

**Affiliations:** 1 Centre for Disease Prevention and Control of Latvia, Rīga, Latvia; 2 Faculty of Medicine, University of Latvia, Riga, Latvia

## Abstract

**Background:**

Ultrasound (US) can help monitor normal fetal development and screen for any potential problems. The prenatal detection of fetal anomalies allows for optimal perinatal management.

**Aim:**

The aim was to assess congenital anomalies at births and their associated antenatal care factors.

**Methods:**

Data source - Health Care Monitoring Datalink (HCMD), including two data sources: Medical Birth Register and ambulatory care data provided by public and private health care providers about US. Screening was detected by specific manipulation code: 50694 - routine US screening in the 1st trimester of pregnancy. All singleton birth in 2018 (n = 12955) were included in the data analysis. OR (odds ratio) were calculated. Multiple regression model was adjusted for mother age, gestational age, living area and antenatal care factors.

**Results:**

The mean mother age was 30.3 (SD 5.4) and gestational age 39.3 (SD 1.8). The use of ICD-10 code O28 - abnormal findings on antenatal screening of mother - was observed in a small number of cases. 2.4% (n = 305) abnormal findings on antenatal screening of mother were detected at ambulatory care visits. From these cases 7.5% (n = 23) were diagnosed congenital anomalies at birth. Totally 2.8% (n = 362) of births were registered congenital anomalies. Congenital anomalies at birth have higher and statistically significant odds of invasive diagnostic methods in pregnancy (OR = 2.0; 95%CI 1.2-3.6; p < 0.01) and abnormal findings on US screening (OR = 2.6; 95%CI 1.7-4.2; p < 0.001). Slightly higher frequency of congenital anomalies at birth but not statistically significant (p > 0.05) were observed for 1st trimester genetic screening (OR = 1.5), preterm deliveries (OR = 1.4) and living in urban area (OR = 1.3).

**Conclusions:**

Pregnancy outcome as congenital anomalies at birth related with higher maternal screening examinations prenatally. Further studies are needed to analyze the efficiency of US examinations for early prenatal detection of congenital anomalies.

**Key messages:**

- Pregnancy outcome as congenital anomalies at birth related with higher maternal screening examinations prenatally. There is not enough information of US screening results in existing data bases.
- Further studies are needed to analyze the efficiency of US examinations for early prenatal detection of congenital anomalies.

